# Poly-D,L-Lactic Acid Filler Increases Hair Growth by Modulating Hair Follicular Stem Cells in Aged Skin

**DOI:** 10.3390/cells15010005

**Published:** 2025-12-19

**Authors:** Seyeon Oh, Jino Kim, Hosung Choi, Hwa Jung Yoo, Kuk Hui Son, Kyunghee Byun

**Affiliations:** 1 Functional Cellular Networks Laboratory, Lee Gil Ya Cancer and Diabetes Institute, Gachon University, Incheon 21999, Republic of Korea; 2 LIBON Inc., Incheon 22006, Republic of Korea; 3 Department of Anatomy & Cell Biology, College of Medicine, Gachon University, Incheon 21936, Republic of Korea; 4 New Hair Plastic Surgery Clinic, Seoul 06134, Republic of Korea; 5 PIENA Aesthetic Medical Clinic, Seoul 06136, Republic of Korea; 6 MODI Hair Plant Clinic, Seoul 06729, Republic of Korea; 7 Department of Thoracic and Cardiovascular Surgery, Gachon University Gil Medical Center, Gachon University, Incheon 21565, Republic of Korea; 8 Department of Health Sciences and Technology, Gachon Advanced Institute for Health & Sciences and Technology (GAIHST), Gachon University, Incheon 21999, Republic of Korea

**Keywords:** poly-D,L-lactic acid, Piezo1, macrophage polarization, hair follicular stem cells

## Abstract

**Highlights:**

**What are the main findings?**

- Poly-D,L-lactic acid (PDLLA) activates Piezo1-mediated mechanotransduction in senescent macrophages, leading to enhanced M2 polarization and increased secretion of growth factors (HGF and IGF-1).
- Conditioned media from PDLLA-treated macrophages promotes hair follicle stem cells (HFSCs) proliferation and stemness via activation of the RAS/ERK and Wnt/β-catenin signaling pathway, resulting in extended anagen phase and increased hair follicle number, diameter, and length in aged mice.

**What are the implications of the main findings?**

- PDLLA rejuvenates the aged skin microenvironment by restoring macrophage function and enhancing the regenerative capacity of HFSCs.
- This study establishes a novel biomaterial-based strategy for treating age-related hair loss, highlighting mechanosensitive ion channel modulation as a promising therapeutic target for hair follicle regeneration.

**Abstract:**

Age-associated hair loss is primarily driven by decreased function and proliferation of hair follicle stem cells (HFSCs), often exacerbated by increased inhibitory signaling and changes in the stem cell niche. Macrophage polarization to the anti-inflammatory M2 phenotype is known to increase stem cell proliferation. We investigated the effects of poly-D,L-lactic acid (PDLLA) on hair growth in middle-aged skin, focusing on its role in modulating macrophage polarization and HFSC activity. Senescent macrophages were analyzed for Piezo1 activity, macrophage polarization, and secretion of hepatocyte growth factor (HGF) and insulin-like growth factor-1 (IGF-1) after PDLLA treatment. Downstream effects on HFSC proliferation, stemness, and Wnt signaling were assessed, including inhibition experiments using the Piezo1 blocker GsMTx4. In vivo analyses assessed hair follicle number, diameter, length, anagen duration, and hair coverage following PDLLA administration in middle-aged mice. PDLLA increased Piezo1 expression and activity in senescent macrophages, enhancing M2 polarization and secretion of HGF and IGF-1. This activated the RAS/ERK signaling pathway, promoting HFSC proliferation and stemness. Furthermore, PDLLA upregulated Wnt signaling molecules (Wnt3a, Wnt10b, and β-catenin) and anagen phase-related factor (Axin2, LEF1, and Lgr5), which were decreased by GsMTX4. In middle-aged animal skin, PDLLA administration led to increased hair follicle number, diameter, and length, as well as prolonged anagen and greater hair coverage. Collectively, these findings suggest that PDLLA rejuvenates the middle-aged skin microenvironment, at least in part through Piezo1-associated M2 macrophage polarization and enhanced HFSC function, offering a promising therapeutic strategy for age-related hair loss targeting both the immune and the stem cell compartments.

## 1. Introduction

Hair follicle stem cells (HFSCs) generate new hair, a process modulated by the dermal papilla (DP) in the hair follicle’s bulb region [1,2,3]. Hair growth cycles through three phases: anagen (growth), catagen (degeneration), and telogen (rest) [1,2,3]. The DP initiates anagen by activating quiescent HFSCs in the bulge region [1,2,3]. Growth factors and cell signals control these transitions. During the resting phase (telogen), the DP secretes inhibitory signals like bone morphogenetic protein (BMP), which keep HFSCs quiescent [1,2,3]. The shift from telogen to anagen is triggered by the activation of HFSCs via Wnt signaling [1,2,3].

Without Wnt signaling, β-catenin is phosphorylated by a ‘destruction complex’ consisting of adenomatous polyposis coli, glycogen synthase kinase 3β (GSK3β), and Axin, leading to its degradation in the cytoplasm [4]. When Wnt binds with Frizzled and lipoprotein receptor-related protein 5/6 receptors, disheveled protein is recruited and inhibits the destruction complex, resulting in increased nuclear translocation of β-catenin [4]. Nuclear β-catenin binds to T cell factor/lymphoid enhancer factor (TCF/LEF) family genes, leading to increased transcription of target genes such as Axin2, LEF1, and Lgr5 [4] and the activation of HFSCs [5], which generate the lower part of the hair follicle [1]. During anagen, Wnt10b and β-catenin levels increase and activate HFSCs [6]. Conversely, Wnt10b and β-catenin levels are decreased during telogen, and decreased β-catenin expression is associated with hair loss [6].

Stem cell niches become altered in aged skin, leading to reduced hair generation, hair thinning, and baldness [7]. In aged mouse skin, senescent HFSCs exhibit decreased colony generation [8], and Wnt inhibitors are increased while BMP inhibitors are decreased [9].

Piezo-type mechanosensitive ion channel component 1 (Piezo1) is a non-selective Ca^2+^-permeable cation channel involved in anti-inflammatory M2 macrophage polarization [10]. Mild mechanical stress stimulates hair regeneration by causing macrophages to adopt the M2 phenotype and secrete hepatocyte growth factor (HGF) and insulin-like growth factor 1 (IGF-1) [11]. IGF-1 upregulates extracellular signal-regulated kinases (ERKs), which are involved in maintaining the stemness of stem cells and controlling hair cycling [12,13]. M2 macrophages also activate Wnt1 and Wnt3, which increase the expression of target genes such as Lgr5 and c-Myc [14].

Poly-D,L-lactic acid (PDLLA) was previously found to regulate Piezo1 and increase M2 polarization and IL-10 secretion, resulting in adipose stem cell proliferation, adipogenesis, and collagen synthesis in aged skin [15,16]. The effects of PDLLA on HFSC proliferation and hair loss have not yet been evaluated. We hypothesized that by increasing Piezo1 activity and M2 polarization, PDLLA treatment would increase HGF and IGF-1 secretion from macrophages, leading to activation of RAS/ERK and increased proliferation of HFSCs. Furthermore, we hypothesized that increased M2 polarization would lead to activation of the Wnt pathway, thus promoting anagen and increased hair growth. We evaluated these hypotheses using middle-aged animal skin and senescent HFSCs and macrophages.

## 2. Materials and Methods

### 2.1. Preparation of PDLLA

PDLLA (VAIM Co., Ltd., Seoul, Republic of Korea) was dissolved in an ethylene carbonate/dimethyl sulfoxide mixture (1:9, *v*/*v*; both from Sigma-Aldrich, St. Louis, MO, USA) and precipitated by spraying into cold n-hexane (−20 °C to −30 °C). The precipitate was washed, dried, and sieved to obtain 10–30 μm particles. These were blended with 0.6% hyaluronic acid (HA; VAIM Co., Ltd.) at a weight ratio of 17:3 (PDLLA:HA), lyophilized, and sterilized with ethylene oxide gas before use [15,17,18].

### 2.2. Laser Diffraction Particle Size Analysis and Rheological Analysis

The particle size distribution of PDLLA microspheres was measured using a laser diffraction particle size analyzer (Mastersizer, Malvern Panalytical, Malvern, UK). Samples were illuminated with a collimated laser beam, and scattered light was collected over a wide angular range by an array of detectors. The laser intensity was automatically adjusted according to sample transmittance to ensure optimal scattering conditions. Particle size distribution parameters, including D10, D50, D90, mean, and mode, were calculated using the Mie scattering model implemented in the manufacturer’s software (version 3.81; Malvern Panalytical).

The rheological properties of the PDLLA suspension were evaluated using a rotational rheometer (ARES-G2, TA instruments, New Castle, DE, USA). Measurements were performed at 25 °C using a 40-mm parallel-plate geometry. Oscillatory shear measurements were conducted at a constant strain of 100% over a frequency range of 0.1–100 Hz, and the storage modulus (G′), loss modulus (G″), and complex viscosity (η*) were recorded and analyzed using the manufacturer’s software (version 5.1; TA instruments).

### 2.3. In Vitro Study

#### 2.3.1. Cell Culture

THP-1 human monocytic cells and human HFSCs were used in this study. THP-1 cells (American Type Culture Collection, #TIB-202, Manassas, VA, USA) were cultured in Roswell Park Memorial Institute 1640 medium (Gibco, Thermo Fisher Scientific, Waltham, MA, USA) supplemented with 10% fetal bovine serum (FBS; Gibco, Thermo Fisher Scientific, Waltham, MA, USA) and 1% penicillin/streptomycin (P/S; Welgene, Gyeongsan, Republic of Korea). HFSCs (HighQC™ Human hair follicle stem cell, AcceGene Biotech, #ABS-SC0097, Fairfield, NJ, USA) were cultured in Human Hair Follicle Stem Cell Complete Growth Medium (AcceGene Biotech, #ABM-SC0097, Fairfield, NJ, USA) according to the manufacturer’s instructions and were supplied as authenticate, quality-controlled cells. All cells were maintained at 37 °C in a humidified incubator with 5% CO_2_.

#### 2.3.2. Induction of Cellular Senescence

Cells exposed to hydrogen peroxide (H_2_O_2_) are commonly used as an in vitro model of senescence, since oxidative stress triggered by hydrogen peroxide reproduces morphological alterations characteristic of senescent cells observed in vivo [19,20,21]. Cellular senescence in THP-1 monocytes and HFSCs was induced by exposure to H_2_O_2_. To obtain adherent macrophage-like cells, non-adherent THP-1 monocytes were first stimulated with 100 ng/mL phorbol 12-myristate 13-acetate (PMA; Sigma-Aldrich, #P8139, St. Louis, MO, USA) for 24 h. Following differentiation, the cells were exposed to 100–400 μM H_2_O_2_ (Sigma-Aldrich, #H1009, St. Louis, MO, USA) 3 h. After H_2_O_2_ treatment, cells were rinsed with Dulbecco’s phosphate-buffered saline (DPBS; Gibco, Thermo Fisher Scientific, Waltham, MA, USA) and maintained in fresh growth medium for an additional 72 h. Cell viability, senescence markers (p16 and p21) [22], and senescence-associated beta-galactosidase (SA-β-gal) activity were measured to identify optimal conditions. Treatment with 100 μM H_2_O_2_ induced cellular senescence without significant cytotoxicity and was used for subsequent experiments.

For HFSCs, H_2_O_2_ was applied at the same concentration range (100–400 μM) under three different treatment protocols: (1) a single 2 h exposure followed by DPBS wash and 72 h incubation in fresh growth medium, (2) repeated 2 h exposures once daily for 3 consecutive days, with DPBS washing followed by replacement with fresh growth medium after each treatment, and (3) a single 3 h exposure followed by DPBS wash and 72 h. After each protocol, cell viability and senescence markers were analyzed. Among the tested conditions, repeated exposure to 100 μM H_2_O_2_ for 2 h per day over 3 days induced senescence without cytotoxicity and was selected as the standard condition for HFSC senescence modeling.

#### 2.3.3. Determination of PDLLA Concentration for Piezo1 Modulation in Senescent Macrophages

To determine the appropriate concentration of PDLLA for modulating Piezo 1 activity, THP-1–derived senescent macrophages were treated with PDLLA across a range of concentrations (0~500 μg/mL). THP-1 cells (1 × 10^6^ cells) were seeded and cultured for 48 h, followed by differentiation into adherent macrophage-like cells using 100 ng/mL PMA for 24 h. Cells were then treated with 100 μM H_2_O_2_ for 3 h to induce senescence, washed with DPBS, and incubated in fresh growth medium for 72 h. After senescence induction, cells were treated with PDLLA at concentrations of 0, 50, 100, 200, 300, 400, and 500 μg/mL. For calcium influx analysis using Fura-2, cells were treated with PDLLA for 2 h. For mRNA expression analysis of Piezo1, cells were treated for 48 h. Cells were then harvested for analysis. Based on the results of Piezo1 activity analysis, 200 μg/mL PDLLA was found to be the most appropriate concentration for modulating Piezo1 in senescent macrophages. This concentration was used for all subsequent experiments.

#### 2.3.4. PDLLA and GsMTx4 Treatment in Senescent Macrophages

Senescent macrophages were divided into four groups: (1) phosphate-buffered saline (PBS), (2) PBS/PDLLA, (3) GsMTx/PBS, and (4) GsMTx4/PDLLA. Cells were pretreated with PBS or GsMTx4 (500 nM; Abcam, #ab141871, Cambridge, UK) for 1 h [23], followed by a 48 h incubation with PBS or PDLLA (200 μg/mL). Culture supernatants were collected, centrifuged, and stored at −80 °C until use.

#### 2.3.5. Treatment of Senescent HFSCs with Conditioned Medium

Senescent HFSCs were exposed 50% (*v*/*v*) CM from senescent macrophage groups diluted in growth medium for 72 h, after which cells were harvested for analysis.

### 2.4. In Vivo Study

#### 2.4.1. Mouse Model and Maintenance

C57BL/6 mice (6-week-old) were originally obtained from Orient Bio (Seongnam, Republic of Korea) and housed under standard laboratory conditions (temperature: 20–24 °C; humidity: 45–55%) with unrestricted access to food and water. Following a 1-week acclimatization period, the mice were bred in-house, and the resulting offspring were maintained and aged for 17 months to allow tissue collection at 18 months of age. All procedures involving animals were conducted in accordance with institutional guidelines and were approved by the Institutional Animal Care and Use Committee of Gachon University (IACUC No. LCDI-2024-0105). Experiments adhered to the ethical standards of the Association for Assessment and Accreditation of Laboratory Animal Care International (AAALAC International, Frederick, MD, USA) and were performed in compliance with the ARRIVE guidelines.

#### 2.4.2. PDLLA Injection for Hair Growth in Middle-Aged Mice

In vivo studies assessing the hair growth-promoting effects of PDLLA were conducted using middle-aged (17 months) female C57BL/6 mice [24]. Animals were anesthetized using 3% isoflurane in oxygen (0.4 L/min), and the dorsal area was shaved using an electric clipper (ISIS, B. Braun, Melsungen, Germany). Depilation was performed in a 2 cm × 2 cm area using cold wax strips (Veet Minima, Reckitt Benckiser, Slough, UK) to synchronize hair follicles in the telogen phase. Residual wax was removed with mineral oil (Sigma-Aldrich), and a protective barrier film (Cavilon; 3M, St. Paul, MN, USA) was applied to prevent skin irritation.

Mice were randomly assigned to three groups (n = 5 per group). Group 1 animals were given 500 μL sterile saline via a single subcutaneous injection; Group 2 received a subcutaneous injection of 500 μL PDLLA suspension (10 mg/mL) and was sacrificed after 1 week; Group 3 received the same PDLLA injection and was sacrificed after 3 weeks. All injections were administered using a 27-gauge needle and divided evenly across five dorsal sites within the depilated area. At the designated time points, mice were sacrificed, and dorsal skin samples were collected for histological analysis.

### 2.5. Cell Viability Assay

To assess the effects of H_2_O_2_ on cell viability, THP-1 cells and HFSCs were treated with H_2_O_2_ as described above. Following the senescence induction protocol, cells were seeded in 96-well plates (SPL Life Sciences, Pocheon, Republic of Korea) and incubated with various concentrations of H_2_O_2_ (0–400 μM). Cell viability was evaluated using the Cell Counting Kit-8 (CCK-8; TransGen Biotech Co., Ltd., #FC101-03, Beijing, China) following the manufacturer’s instructions. The absorbance was measured at 450 nm with a microplate reader, and all assays were conducted in triplicate.

### 2.6. RNA Isolation and Quantitative Gene Expression Analysis

RNA extraction was carried out using RNAiso reagent (Takara Bio Inc., #9708, Kusatsu, Japan) according to the manufacturer’s instructions. RNA concentration and purity were evaluated with a NanoDrop spectrophotometer (Thermo Fisher Scientific, Waltham, MA, USA). Complementary DNA (cDNA) was synthesized from 1 μg of RNA using a reverse transcription kit (Takara Bio Inc., #6121, Kusatsu, Japan) following the manufacturers’ recommendations.

Quantitative real-time PCR (qRT-PCR) was performed using SYBR Green chemistry (Takara Bio Inc., #RR82LR, Kusatsu, Japan) on a QuantStudio™ 3 Real-Time PCR System (Applied Biosystems, Thermo Fisher Scientific, Waltham, MA, USA). Amplification conditions consisted of an initial denaturation at 95 °C for 10 min, followed by 40 cycles of 95 °C for 15 s and 60 °C for 1 min. Relative gene expression was determined using the comparative Ct (ΔΔCt) method, with ACTB (β-actin) used as the internal control. The primer sequences used for each target gene are provided in Table S1.

### 2.7. SA-β-Galactosidase Staining

Senescence-associated β-galactosidase activity was examined with a commercial staining kit (Cell Signaling Technology, #9860, Danvers, MA, USA) following the supplier’s recommendations. After rinsing the cells with PBS, they were fixed for 15 min at room temperature and then exposed to a staining solution adjusted to pH 6.0. The plates were placed in a CO_2_-free incubator at 37 °C and left overnight. On the next day, cells displaying blue coloration under a light microscope were regarded as SA-β-gal–positive. For analysis, images from at least five randomly chosen fields per sample were acquired, and the fraction of positive cells was calculated.

### 2.8. Intracellular Calcium Measurement Using Fura-2

Intracellular calcium levels were quantified using the radiometric fluorescent dye Fura-2 AM (Thermo Fisher Scientific, #F1225, Waltham, MA, USA) and a fluorescence microplate reader. Cells were seeded in 96-well plates and incubated with 5 μM Fura-2 AM in loading buffer (HBSS containing 1 mM CaCl_2_ and 0.04% pluronic acid) at 37 °C for 30 min in the dark. After washing with HBSS, fluorescence was measured using a microplate reader (SpectraMax iD5, Molecular Devices, San Jose, CA, USA) with excitation wavelengths of 340 and 380 nm and emission at 510 nm. The ratio of fluorescence intensity at 340/380 nm was used to quantify the intracellular calcium concentration. All measurements were performed in triplicate.

### 2.9. Flow Cytometry Analysis of Macrophage Polarization Markers

Flow cytometry was used to characterize the polarization status of THP-1–derived macrophages by monitoring the surface levels of CD86 (M1-associated) and CD206 (M2-associated). After the PDLLA and GsMTx4 treatment, senescent macrophages were detached with trypsin-EDTA (Thermo Fisher Scientific, Waltham, MA, USA), collected, and then washed twice in fluorescence-activated cell sorting (FACS) buffer consisting of PBS with 1% FBS). For polarization controls, TH-1–derived macrophages were stimulated with 20 ng/mL IFN-γ (Sigma-Aldrich, #SRP3058, St. Louis, MO, USA) plus 10 pg/mL LPS (Sigma-Aldrich, #L2630, St. Louis, MO, USA; M1 condition) or 20 ng/mL IL-4 (Sigma-Aldrich, #SRP3093, St. Louis, MO, USA; M2 condition) for 24 h [25] prior to staining and were used to define M1 and M2 gating. For staining, 1 × 10^5^ cells per tube were resuspended in FACS buffer and incubated for 30 min at 4 °C in the dark with anti-CD86 (1 μg/1 × 10^5^ cells; Santa Cruz Biotechnology, #sc-19617, Westminster, CO, USA), anti-CD206 (1 μg/1 × 10^5^ cells; Novus Biogicals LLC, #NBP1-90020, Dallas, TX, USA) and Alexa 594 or 488–conjugated secondary antibodies (5 μg/1 × 10^5^ cells; Invitrogen, Thermo Fisher Scientific, Waltham, MA, USA). Appropriate isotype controls were included to set gating thresholds.

After labeling, cells were washed again, resuspended in FACS buffer, and analyzed on a BD FACS Calibur flow cytometr (BD Biosciences, Franklin Lakes, NJ, USA) equipped with standard fluorescent filters. At least 10,000 events were acquired for each sample, and results were reported as mean fluorescence intensity and as the proportion of CD86^+^ or CD206^+^ cells.

### 2.10. Protein Isolation and Quantitation

Total proteins were extracted from cultured cells and skin tissue using EzRIPA buffer (ATTO Corporation, #WSE-7420, Tokyo, Japan). Protein levels in the lysates were quantified with a bicinchoninic acid assay (Thermo Fisher Scientific, #23225, Waltham, MA, USA). The resulting lysates were either processed immediately for subsequent experiments or aliquoted and stored at −80 °C until use.

### 2.11. Western Blot

For Western blot, 30 μg of protein from each cells or skin lysates was combined with 4× lithium dodecyl sulfate sample buffer (Invitrogen, Thermo Fisher Scientific, #NP0007, Waltham, MA, USA) and 10× reducing reagent (Invitrogen, Thermo Fisher Scientific, #NP0009, Waltham, MA, USA), followed by denaturation at 70 °C for 10 min. Denatures samples were resolved on 10% sodium dodecyl sulfate–polyacrylamide gels and in MOPS running buffer (Invitrogen, Thermo Fisher Scientific, #NP0001, Waltham, MA, USA) at 200 V for 25 min and then transferred to polyvinylidene fluoride membranes (Millipore, #MILL-IPVH00010, Burlington, MA, USA) using a semi-dry blotting apparatus (ATTO, Tokyo, Japan) operated at 1 A for 10 min. The membranes were blocked for 1 h at room temperature on a shaker in Tris-buffered saline containing 0.1% Tween 20 (TTBS; SPL Life Sciences, Pocheon, Republic of Korea) supplemented with 5% skim milk (LPS solution, Daejeon, #SKI500, Republic of Korea). After three rinses with 0.1% TTBS, membranes were incubated overnight at 4 °C with primary antibodies diluted in the same buffer (see Table S2). Subsequently, horseradish peroxidase (HRP)–conjugated secondary antibodies (1:5000; Vector Laboratories, Burlingame, CA, USA) were applied for 1 h at room temperature, followed by additional TTBS washes. Immune complexes were detected using enhanced chemiluminescence substrates and capture on a ChemiDoc imaging system (Bio-Rad, Hercules, CA, USA). Band densities were measured with ImageJ software (version 1.53s; NIH, Bethesda, MD, USA), using β-actin as the loading control, and signal intensities were normalized to the corresponding group.

### 2.12. Enzyme-Linked Immunosorbent Assay (ELISA)

Flat-bottom 96-well plates (Nunc MaxiSorp, Thermo Fisher Scientific, Waltham, MA, USA) were first coated at 4 °C overnight with diluted samples prepared in carbonate–bicarbonate coating buffer (pH 9.6). The next day, residual binding sites were blocked for 1 h at room temperature using PBS containing 5% skim milk, after which the wells were incubated for 2 h with a primary antibody (Table S2) diluted in the same blocking buffer. Plates were then washed with TPBS (PBS supplemented with 0.1% Tween-20), and HRP–conjugated secondary antibodies (Vector Laboratories, Burlingame, CA, USA) were applied for 1 h at room temperature. Color was developed by adding 3,3′,5,5′-tetramethylbenzidine (TMB) substrate solution (Sigma-aldrich, #54827-17-7, St. Louis, MO, USA), and the enzymatic reaction was terminated with 1 M sulfuric acid. Absorbance was read at 450 nm using a Multiskan SkyHigh microplate spectrophotometer (Thermo Fisher Scientific, Waltham, MA, USA).

### 2.13. Fixation and Paraffin-Embedding of Skin Tissue

Skin specimens were immersed in 4% paraformaldehyde (Sigma-Aldrich, #158127, St. Louis, MO, USA) for 72 h, processed through graded dehydration, and infiltrated with paraffin in an automated tissue processor (Leica, Wetzlar, Germany). The resulting paraffin blocks were cut into 7-µm sections on a microtome, placed onto glass slides, and baked overnight at 60 °C to ensure firm adhesion.

### 2.14. Hematoxylin and Eosin Staining

Paraffin sections of skin were first deparaffinized in xylene and then rehydrated by passage through a descending ethanol series (100–70%). After rehydration, slides were incubated with hematoxylin (KPNT, #SH-001, Cheongju, Republic of Korea) for 1 min, rinsed in tap water, and subsequently counterstained with eosin (KPNT, #SE-001, Cheongju, Republic of Korea) for 30 s. Stained sections were dehydrated, coverlipped, and scanned using a slide scanner (Motic Scan Infinity 100; Motic, Beijing, China) for image acquisition, Hair follicle number, diameter and length were measured from the scanned images using ImageJ software (version 1.53s, NIH, Bethesda, MD, USA).

### 2.15. Immunohistochemistry (DAB, 3,3′-Diaminobenzidine)

After deparaffinization, tissue sections were incubated for 1 h at room temperature in a a serum-based blocking solution (Vector Laboratories, Burlingame, CA, USA) to minimize nonspecific binding. Slides were then exposed overnight at 4 °C to the primary antibodies listed in Table S2. On the following day, sections were rinsed with PBS and incubated for 1 h at room temperature with biotinylated secondary antibodies (1:200; Vector Laboratories, Burlingame, CA, USA). After an additional PBS wash, the VECTASTAIN ABC reagent (Vector Laboratories, #PK-6100, Burlingame, CA, USA) was applied according to the manufacturer’s direction, followed by another wash step. Chromogenic signal was developed by treating the sections with 3,3′-diaminobenzidine (DAB) solution (Sigma-Aldrich, #D7304, St. Louis, MO, USA) for approximately 30 s until a brown reaction product was evident. Slides were then counterstained with hematoxylin (KPNT, Cheongju, Republic of Korea) for 30 s, rinsed in distilled water, dehydrated through graded ethanol, and coverslipped using DPX mounting medium (Sigma-Aldrich, #06522, St. Louis, MO, USA). Stained sections were digitized with a slide scanner (Motic Scan Infinity 100; Motic, Beijing, China), and images were used for quantitative analysis. For quantification of protein expression, DAB-positive areas within skin or hair follicle regions were delineated in ImageJ (version 1.53s, NIH, Bethesda, MD, USA) using color thresholding. The mean staining intensity within each selected region was measured, and values from five randomly chosen dermal fields per group were used for statistical analysis.

### 2.16. Immunofluorescence

Paraffin–embedded skin sections (4 μm thick) were processed for immunofluorescence staining. Sections were first deparaffinized in xylene and then rehydrated through a descending ethanol series. Antigen retrieval was carried out by incubating the slides in 10 mM citrate buffer (pH 6.0) at 95 °C for 20 min, followed by cooling to room temperature. The sections were rinsed in PBS and permeabilized for 10 min with 0.5% Triton X-100 in PBS, after which nonspecific binding sites were blocked with 5% normal serum for 1 h at room temperature.

Primary antibodies listed in Table S2, diluted in blocking buffer, were applied to the sections and allowed to bind overnight at 4 °C in a humidified chamber. After PBS washes, Alexa Fluor–conjugated secondary antibodies (Thermo Fisher Scientific, Waltham, MA, USA) were added for 1 h at room temperature in the dark. Nuclei were labeled with 4′,6-diamidino-2-phenylindole (DAPI, 1 μg/mL; Sigma-Aldrdich, #D9542, St. Louis, MO, USA), and slides were mounted in antifade mounting medium (Vector Laboratories). Fluorescence images were collected on an LSM-710 confocal microscopy (Carl Zeiss, Oberkochen, Germany) at the Core-facility for Cell to in vivo imaging. For each experimental group, five randomly chosen fields were analyzed, and the number of double-stained cells (K15^+^/PCNA^+^) were quantified using Zen imaging software (version 5.1; Carl Zeiss, Oberkochen, Germany).

### 2.17. Hair Coverage Analysis

To evaluate macroscopic hair regrowth, the depilated dorsal skin of mice was photographed at a fixed distance under standardized lighting on weeks 0 (initial), 1, and 3 after injection, using the same positioning and camera setting for all animals. For each image, a region of interest corresponding to the depilated dorsal area was defined, excluding the head and limbs. To quantify hair coverage, dorsal images were processed using ImageJ software (version 1.53s, NIH, Bethesda, MD, USA). After converting images to grayscale, a uniform threshold value was applied to segment hair-covered regions, and binary masks were generated. The percentage of the hair-covered area was then calculated within the defined region of interest and used as a quantitative index of hair coverage [26,27].

### 2.18. Statistical Analysis

Sample sizes (n) for each experiment are specified in the figure legends. In vitro assays were performed using three independent biological replicates, and in vivo studies used five mice per group. Quantitative data are presented as the mean ± standard deviation. Statistical comparisons were performed using the Kruskal–Wallis test followed by the pairwise Mann–Whitney U test for post hoc analysis. Different letters indicate significant differences between groups (*p* < 0.05). All statistical analyses were performed using SPSS version 26 (IBM, Armonk, NY, USA).

## 3. Results

### 3.1. Physical Characterization of PDLLA

Laser diffraction analysis confirmed that the PDLLA preparation consisted of micro-scale particulate populations, with a predominant peak centered around 23.5 μm (Dv10 = 13.3 μm, Dv50 = 23.5 μm, Dv90 = 39.7 μm). Rheological characterization demonstrated that PDLLA exhibited measurable viscoelastic properties, with a storage modulus (G′) of 0.017 Pa, a loss modulus (G″) of 0.026 Pa, and a complex viscosity of 0.005 Pa·s at 1 Hz at 25 °C. Steady-shear flow measurements showed an approximately constant viscosity (~0.004 Pa·s) at shear rates of 10–100 s^−1^. Together, these data confirm that the PDLLA formulation comprises microscale particulates suspended in a weakly viscoelastic medium. Such particulate microenvironments can expose macrophages to localized mechanical interaction at the cell-particle interface [28,29]. Although the bulk storage modulus of the PDLLA suspension is low, the presence of rigid PDLLA microspheres with micrometer-scale curvature is expected to generate localized membrane deformation upon cell-particle contact [29,30], providing a plausible mechanical context for activation of mechanosensitive pathways, including Piezo1, considered in this study.

### 3.2. PDLLA Increases M2 Polarization and Secretion of HGF and IGF-1 in Senescent Macrophages

To generate a model of macrophage senescence, we treated THP-1 cells with H_2_O_2_ at concentrations ranging from 0 to 400 μM. Cell viability was significantly reduced at concentrations ≥ 200 μM, indicating cytotoxicity at higher doses (Figure S1A). Oxidative stress, measured by 8-hydroxy-2′-deoxyguanosine (8-OHdG) levels [31], was elevated in cells treated with ≥100 μM H_2_O_2_ (Figure S1B). Levels of the senescence-associated markers *P21* and *P16* were also upregulated by 100 μM H_2_O_2_ (Figure S1C,D). Consistently, the number of SA-β-galactosidase–positive cells was increased in the presence of 100 μM H_2_O_2_ (Figure S1E,F). Based on these results, we selected 100 μM H_2_O_2_ for subsequent experiments.

To determine the optimal concentration of PDLLA for in vitro experiments, we treated senescent macrophages with various concentrations of PDLLA. *Piezo1* mRNA expression was increased starting at 100 μg/mL PDLLA and reached a maximum at 200 μg/mL PDLLA (Figure S2A). Intracellular Ca^2+^ influx was most prominently elevated at 200 μg/mL PDLLA (Figure S2B). The optimal concentration of PDLLA was thus determined to be 200 μg/mL, based on its maximal induction of Piezo1 activity.

**Figure 1:**
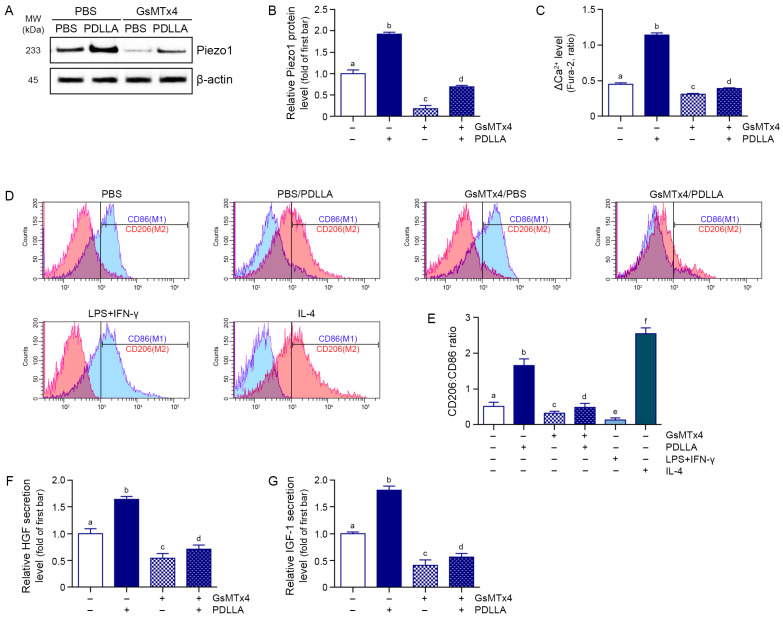
PDLLA-induced Piezo1 activation promotes M2 polarization and growth factor secretion in senescent macrophages. (**A**) Western blot analysis of Piezo1 expression in senescent macrophages treated with PBS or PDLLA, with or without GsMTx4 (a Piezo1 inhibitor). β-actin was used as a loading control; (**B**) Densitometric quantification of Piezo1 protein levels normalized to β-actin and presented as fold change relative to the PBS group (first bar); (**C**) Intracellular calcium (Ca^2+^) influx was evaluated using Fura-2 fluorescence ratio (340/380 nm) after PDLLA treatment, in the presence or absence of GsMTx4; (**D**) Representative flow cytometry histograms showing CD86 (M1 marker, blue) and CD206 (M2 marker, red) expression in senescent macrophages treated with PBS, PDLLA, GsMTx4 or PDLLA + GsMTx4, along with LPS + IFN-γ (M1 control) and IL-4 (M2 control); (**E**) Quantification of the CD206:CD86 ratio corresponding to the conditions shown in (**D**); (**F**,**G**) Secreted levels of HGF (**F**) and IGF-1 (**G**) in culture supernatant were measured by ELISA. Data are presented as the mean ± SD (n = 3 per group). Statistical significance was determined using the Kruskal–Wallis test followed by pairwise Mann–Whitney U tests. Group labeled with different letters differ significantly (*p* < 0.05).

To investigate whether PDLLA induces M2 polarization by activating Piezo1, we treated senescent macrophages with PDLLA in the presence or absence of the Piezo1 inhibitor GsMTx4. PDLLA treatment increased Piezo1 protein expression by 1.9-fold, which was suppressed by GsMTx4 co-treatment (Figure 1A,B). PDLLA also increased intracellular Ca^2+^ influx by 2.5-fold compared to PBS treatment, whereas GsMTx4 inhibited this response (Figure 1C). PDLLA increased the CD206 (M2 macrophage marker):CD86 (M1 macrophage marker) ratio by 3.2-fold, which was decreased by GsMTx4. In addition, inclusion of canonical M1 (LPS + IFN-γ) and M2 (IL-4) controls confirmed that PDLLA-induced polarization shifted macrophages toward an M2-like phenotype (Figure 1D,E). Moreover, PDLLA treatment increased HGF and IGF-1 secretion by 1.6-fold and 1.8-fold, respectively, which was suppressed by co-treatment with GsMTx4 (Figure 1F,G). These results suggest that PDLLA enhances M2 polarization through Piezo1 activation, leading to increased secretion of HGF and IGF-1.

### 3.3. PDLLA Increases HFSC Proliferation and Cell Signals Related to Anagen

To establish a model of HFSC senescence, we treated HFSCs with H_2_O_2_ under various conditions. When H_2_O_2_ was applied for 3 h, the cell survival rate was low, so this condition was excluded (Figure S3A). The condition that produced the greatest increases in 8-OHdG, *P21*, *P16*, and SA-β-gal was the three repeated exposures to 100 μM H_2_O_2_ (Figure S3B–E), so we used these conditions for subsequent experiments.

**Figure 2:**
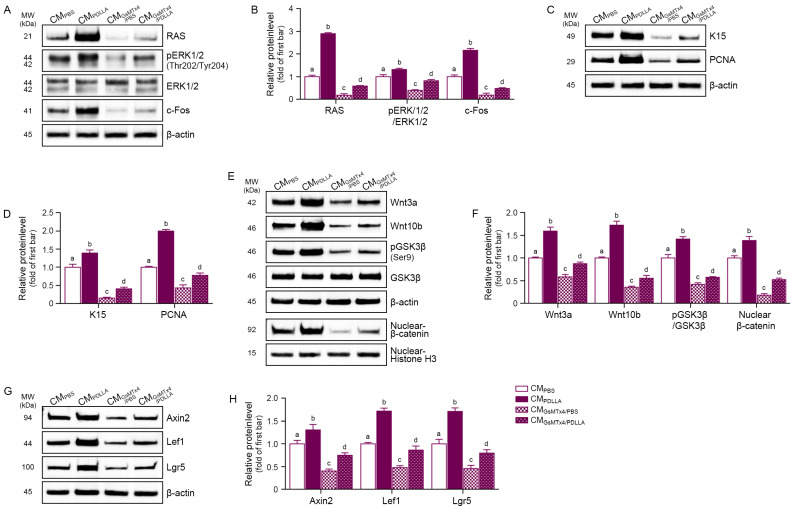
Conditioned medium from PDLLA-treated macrophages activates HFSCs via the RAS/ERK and Wnt/β-catenin signaling pathways. (**A**,**B**) Western blot analysis (**A**) and quantification (**B**) of RAS, phosphorylated ERK1/2 (pERK1/2), total ERK1/2, and c-Fos protein levels in senescent HFSCs treated with conditioned media (CM) derived from PBS-, PDLLA-, GsMTx4-, or PDLLA/GsMTx4-treated macrophages (designated as CM_PBS_, CMP_DLLA_, CM_GsMTx4/PBS_, and CM_GsMTx4/PDLLA_, respectively); (**C**,**D**) Expression levels of proliferation and stemness markers K15 and PCNA were evaluated by Western blot (**C**) and quantified (**D**); (**E**,**F**) Protein levels of Wnt3a, Wnt10b, GSK3β, phosphorylated GSK3β (pGSK3β), and nuclear β-catenin were assessed by Western blot (**E**) and quantified (**F**); (**G**,**H**) Western blot analysis (**G**) and densitometric quantification (**H**) of Wnt/β-catenin target proteins Axin2, Lef1, and Lgr5. Band intensities were normalized to β-actin for total lysates and to histone H3 for nuclear fractions, which served as respective loading controls, and are expressed as fold change relative to the CM_PBS_ group (first bar). Data are presented as the mean ± SD (n = 3 per group). Statistical differences among groups were assessed using the Kruskal–Wallis test followed by pairwise Mann–Whitney U tests. Bars annotated with different letters denote statistically significant differences between groups (*p* < 0.05).

We hypothesized that HFSC proliferation is promoted by HGF and IGF-1 secreted by M2 macrophages, so we treated senescent HFSCs with conditioned media (CM) from senescent macrophages previously treated with PBS, PDLLA, GsMTx4, or PDLLA/GsMTx4. We found that RAS/ERK signaling, which upregulates HFSC proliferation via c-Fos activation [32], was enhanced by CM from PDLLA-treated macrophages (CM_PDLLA_) but reduced by CM from GsMTx4-treated macrophages (CM_GsMTx4_; Figure 2A,B). K15, a marker of stemness in the hair follicle [33], and proliferating cell nuclear antigen (PCNA), a proliferation marker [34], were upregulated by CM_PDLLA_ (1.39-fold and 2.0-fold, respectively) and suppressed by CM_GsMTx4_ (Figure 2C,D). Anagen induction markers, such as Wnt3a and Wnt10b, GSK3β phosphorylation at Ser9, and nuclear β-catenin, were increased by CM_PDLLA_ and decreased by CM_GsMTx4_ (Figure 2E,F). Expression of β-catenin downstream targets, including Axin2, LEF1, and Lgr5, was also elevated in response to CM_PDLLA_ treatment (1.30-fold, 1.72-fold and 1.71-fold, respectively) and decreased by CM_GsMTx4_ (0.57-fold, 0.50-fold and 0.47-fold, respectively; Figure 2G,H). These results suggest that PDLLA promotes anagen induction and maintenance in senescent HFSCs through mechanisms associated with increased Piezo1 expression, enhanced stemness, and proliferation.

### 3.4. PDLLA Increases Piezo1 Expression and M2 Polarization in Middle-Aged Animal Skin

**Figure 3:**
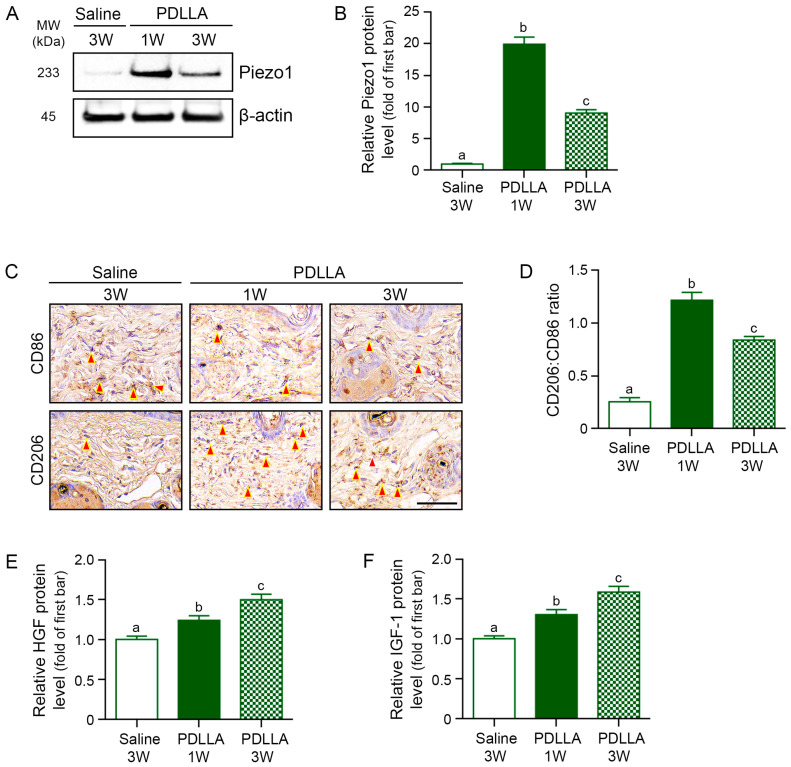
PDLLA enhancesPiezo1 expression, M2 macrophage polarization, and growth factor production in middle- aged mouse skin. (**A**,**B**) Western blot analysis (**A**) and densitometric analysis (**B**) of Piezo1 in dorsal skin from middle-aged mice treated with saline or PDLLA for 1 or 3 weeks. Signals were normalized to β-actin and expressed as fold change relative to the saline group (first bar); (**C**) Representative immunohistochemical staining for CD86 (M1 marker) and CD206 (M2 marker), red arrowheads denote positive cells. Scale bar = 100 μm; (**D**) CD206:CD86 ratio derived from immunohistochemistry; (**E**,**F**) Tissue levels of HGF (**E**) and IGF-1 (**F**) determined by ELISA and plotted relative to saline-treated controls (first bar). Value represent mean ± SD (n = 5 per group). Group differences were evaluated with a Kruskal–Wallis test followed by pairwise Mann–Whitney U tests. Bars bearing distinct letters differ significantly at *p* < 0.05.

PDLLA increased Piezo1 protein expression by 19.91-fold at week 1 and by 9.04-fold at week 3 compared with saline-injected controls (Figure 3A,B). PDLLA also increased the ratio of CD206 (an M2 macrophage marker) to CD86 (an M1 macrophage marker) by 4.77-fold at week 1 and 3.30-fold at week 3 in the middle-aged skin (Figure 3C,D). Furthermore, PDLLA injection increased HGF protein levels by 1.24-fold at week 1 and by 1.50-fold at week 3, and IGF-1 protein levels by 1.30-fold at week 1 and by 1.59-fold at week 3, compared with saline controls (Figure 3E,F).

### 3.5. PDLLA Activates RAS/ERK and Wnt/β-Catenin Signaling and Promotes HFSC Activation in Middle-Aged Animal Skin

**Figure 4:**
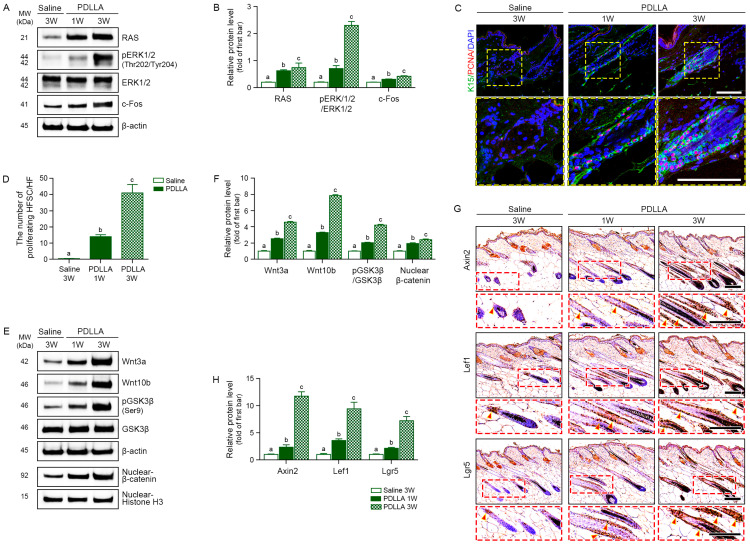
PDLLA activates RAS/ERK and Wnt/β-catenin signaling and enhances hair follicle regeneration in middle-aged mouse skin. (**A**,**B**) Western blot (**A**) and densitometric analysis (**B**) of RAS, phosphorylated ERK1/2 (pERK1/2), total ERK1/2, and c-Fos in dorsal skin harvested 1 or 3 weeks after PDLLA injection. Band intensities were normalized to β-actin and expressed as fold change versus the saline group (first bar); (**C**) Immunofluorescence images of K15 (green) and PCNA (red) in the hair follicle bulge; nuclei are counterstained with DAPI (blue). Insets depict magnified views. Scale bar = 100 μm; (**D**) Quantification of proliferating PCNA^+^ cells within K15^+^ hair follicle (HF) bulge regions. PCNA markers proliferating cells, and K15 identifies hair follicle stem cells (HFSC). The number of PCNA^+^ cells within K15^+^ bulge regions was quantified per field in saline group and PDLLA injected groups at 1 and 3 weeks; (**E**,**F**) Western blot (**E**) and densitometric analysis (**F**) of Wnt3a, Wnt10b, GSK3β, pGSK3β, and nuclear β-catenin. Protein signals were normalized to β-actin for total lysates and histone H3 for nuclear fractions, serving as loading controls, and are shown as fold change relative to saline-injected skin (first bar); (**G**,**H**) Immunohistochemical staining (**G**) and DAB signal quantification (**H**) for Axin2, Lef1, and Lgr5 in hair follicles, red arrowheads mark positive cells (brown). Scale bar = 100 μm. Values are reported as the mean ± SD (n = 5 per group). Group differences were tested using the Kruskal–Wallis with post hoc pairwise Mann–Whitney U tests. Bars annotated with distinct letters represent group that differ significantly (*p* < 0.05).

PDLLA injection increased the expression of RAS, phosphorylated ERK1/2 (pERK1/2), total ERK1/2, and c-Fos in middle-aged skin, with greater induction observed at 3 week (3.71-fold, 11.52-fold and 2.10-fold, respectively) compared with 1 week (3.13-fold, 3.55-fold and 1.54-fold, respectively; Figure 4A,B).

Immunofluorescence staining revealed that PDLLA substantially increased the numbers of PCNA^+^ cells in the K15^+^ follicle bulge region, by 30.11-fold at week 1 and 88.17-fold at week 3, indicating enhanced HFSC proliferation (Figure 4C,D). Key regulators of the Wnt/β-catenin signaling pathway, including Wnt3a, Wnt10b, phosphorylated GSK3β (Ser9), and nuclear β-catenin, were upregulated by PDLLA in a time-dependent manner (Figure 4E,F). In line with these findings, expression of the downstream Wnt/β-catenin targets Axin2, Lef1, and Lgr5 was also increased by PDLLA in a time-dependent manner (Figure 4G,H).

**Figure 5:**
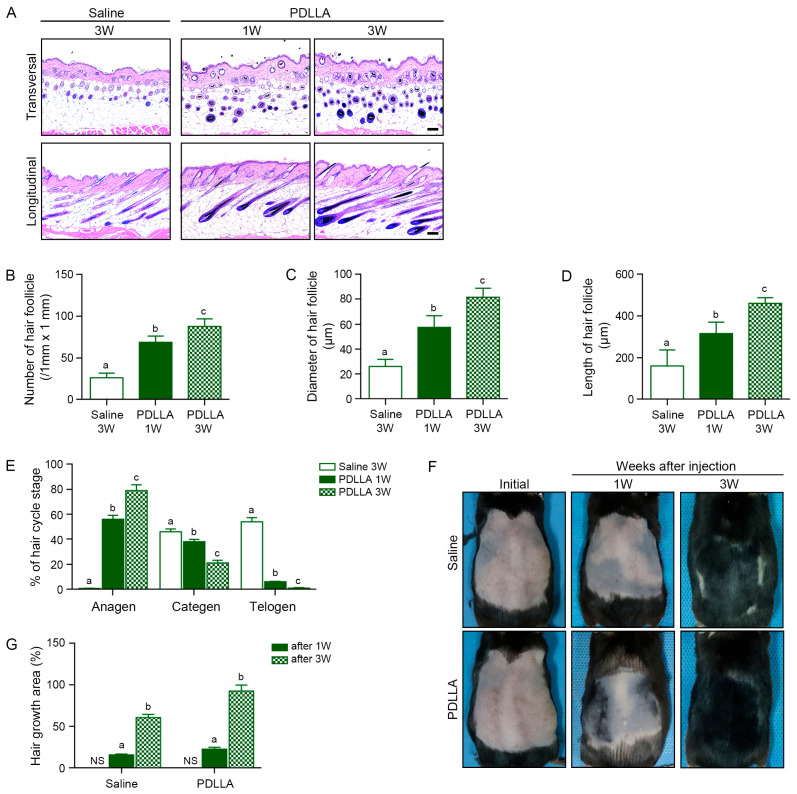
PDLLA injection enhances hair follicle structure and promotes hair growth in middle-aged mouse skin. (**A**) Representative hematoxylin and eosin (H&E)-stained transverse and longitudinal sections of dorsal skin from the saline or PDLLA for 1 or 3 weeks. Images show changes in hair follicle number and morphology. Scale bar = 100 μm; (**B**–**D**) Quantification of hair follicle parameters, including the number (**B**), diameter (**C**), and length (**D**) of hair follicles, following treatment; (**E**) Proportional distribution of hair follicle phases (anagen, catagen, telogen) assessed at 3 weeks post-injection. PDLLA-treated skin showed a significant shift toward the anagen phase; (**F**) Representative photographs of dorsal skin showing progressive hair regrowth at 1 and 3 weeks after following PDLLA injection; (**G**) Quantification of hair growth area (%) at the initial time point and at 1 and 3 weeks after injection in saline- and PDLLA-injected middle-aged mice, based on the dorsal skin images in (**F**). Values are shown as mean ± SD (n = 5 per group). Statistical comparisons among groups were carried out using a Kruskal–Wallis test with post hoc Mann–Whitney U tests for pairwise contrasts. Columns marked with non-identical letters indicate significant differences between groups (*p* < 0.05).

The number of hair follicles was increased by PDLLA in the dorsal skin of middle-aged mice, with greater effects observed at 3 weeks (3.35-fold) compared with 1 week (2.63-fold; Figure 5A,B). PDLLA also increased the diameter and length of the hair follicles, by 2.20-fold at week 1 and 3.12-fold at week 3 (Figure 5A,C,D). Hair cycle staging based on morphological criteria demonstrated a marked increase in the proportion of follicles in anagen, accompanied by reductions of follicles in catagen and telogen, in PDLLA-injected mice (Figure 5A,E). Gross examination of the dorsal skin revealed an increase in visible hair coverage over time, further confirming the promotion of hair regrowth by PDLLA (Figure 5F). Quantitative analysis confirmed that hair-covered area increased over time in both saline- and PDLLA- injected middle-aged mice; however, the increase was significantly greater in PDLLA-injected mice at both week 1 (1.46-fold) and week 3 (1.53-fold) compared with the corresponding saline controls (Figure 5G).

## 4. Discussion

Although hair loss is not considered to have a profound effect on survival, it reduces quality of life and psychological well-being [35]. Aging induces hair loss. Increased oxidative stress and inflammation lead to HFSC dysfunction, which affects the hair cycle [36]. In aged mouse skin, anagen is shortened and telogen is increased, leading to hair thinning and loss [8,37]. To reduce age-related hair loss, it is essential to restore HFSC function and hair cycle progression. Previously, our group showed that PDLLA modulates adipose stem cells by promoting M2 macrophage polarization [15,16]. M2 macrophages secrete anti-inflammatory cytokines [38] and are involved in stem cell proliferation and self-renewal [39].

Piezo1 is a mechanosensitive ion channel activated by changes in cell membrane tension [10,29]. Piezo1 activity is influenced by surface stiffness [29]. Macrophages are among the first cells to respond to surgical implantation of materials, and they are highly sensitive to mechanical cues. Hence, changes in tissue stiffness and immune responses following surgical implantation likely correlate with alterations of Piezo1 activity in macrophages [29]. In one study, mechanical stretching resulted in M2 macrophage polarization via Piezo1 activation, which led to increased proliferation of bone marrow mesenchymal stem cells and osteogenic differentiation [10].

We used senescent HFSCs and macrophages to explore the mechanisms by which PDLLA reduces hair loss related to HFSC aging. Previous studies have employed naturally aged animals to study HFSC aging [37]; however, in vitro models of HFSC senescence have rarely been used. Oxidative stress induced by H_2_O_2_ is widely used in in vitro senescence models [40], so we treated HFSCs with H_2_O_2_ to induce senescence, which we confirmed by evaluating senescence markers to ensure the model’s relevance. In our experiments with senescent macrophages, we found that PDLLA treatment increased Piezo1 expression and activity, as measured by Ca^2+^ influx, and it also enhanced HGF and IGF-1 secretion. By contrast, treatment with the Piezo1 inhibitor GsMTx4 markedly reduced both PDLLA-induced Piezo1 activity and M2 polarization of senescent macrophages. These findings suggest that PDLLA can rejuvenate senescent macrophages by promoting mechanotransduction pathways involving Piezo1, although we cannot exclude a contribution from other GsMTx4-sensitive channels, thereby creating a conducive microenvironment for hair regeneration.

Stem cells have capacities for self-renewal and multipotent differentiation [41]. These stem cell capacities are reduced during natural aging [42] and during long-term in vitro culture [43]. Upregulation of FGF signaling pathways and the RAS/ERK axis has been suggested as a way to maintain stemness [42]. IGF-1 was shown to be involved in the maintenance of stemness [12], and IGF-1 and HGF increased β-catenin levels by activating the PI3k-Akt-GSK3β pathway [12,13]. Our results show that PDLLA increased stemness marker (K15) expression, HFSC proliferation, and GSK3β and β-catenin levels. Depending on the phosphorylation site, GSK3β differentially affects β-catenin stabilization. Phosphorylation at Ser9 leads to GSK3β inactivation, which increases β-catenin stabilization [44]. M2 macrophage polarization was previously reported to increase Wnt3a expression [14]. Our results show that PDLLA increases the expression of Wnt3a and Wnt10b. Thus, PDLLA increases both Wnt expression and β-catenin stabilization, which could increase the transcription of genes involved in anagen.

The pathways that were increased by PDLLA were decreased by GsMTx4. GsMTx4 is frequently used as a Piezo1 inhibitor; however, it may block other channels such as Transient receptor potential channel (TRPC)6 [45]. Like Piezo1, TRPC6 can increase Ca^2+^ influx in response to mechanical stresses such as stretching [46]. Piezo1 can also affect transient receptor potential channel subfamily V member (TRPV)4 activity [47], which was reported to be involved in macrophage polarization [48]. Because we inhibited Piezo1 using the non-selective inhibitor GsMTx4, it is unclear whether PDLLA increased M2 polarization solely by upregulating Piezo1, or whether it did so through complex interactions with TRPC6 or TRPV4. To clarify whether increased M2 polarization induced by PDLLA is due solely to the action of Piezo1, future research should employ methods like Piezo1 deletion. In particular, future studies will complement the pharmacological inhibition used here with genetic approaches such as Piezo1 knockdown (e.g., siRNA/shRNA) or CRISPR/Cas9-mediated deletion in macrophages and/or HFSCs to definitively dissect the channel-specific contribution. Furthermore, we did not investigate the mechanism by which PDLLA increases Piezo1 activity. We speculate that PDLLA injection alters tissue stiffness, and this mechanical cue influences macrophage Piezo1 expression or activity. Future research should aim to elucidate the precise mechanisms by which PDLLA enhances Piezo1 activity.

Similar to the results of our in vitro study, PDLLA increased Piezo1, HGF, and IGF-1 expression and M2 polarization in middle-aged animal skin. RAS, ERK, c-Fos, K15, PCNA, Axin2, Lef1, Lgr5, and Wnt expression (Wnt3 and Wnt10b), GSK3β phosphorylation at Ser9, and nuclear β-catenin levels were also increased by PDLLA. These changes were accompanied by increased hair growth and increased follicle density and size in middle-aged skin. Treatments for alopecia differ depending on the type. Minoxidil or finasteride can reduce follicle atrophy in androgenetic alopecia [49], whereas corticosteroids or Janus kinase (JAK) inhibitors are used for alopecia areata [50,51]. Different from those types of alopecia, age-induced hair loss is mainly associated with dysfunctional HFSCs. To rejuvenate HFSCs during aging, JAK inhibitors such as pyridine 6 have been tested for their ability to reduce age-related inflammation. When pyridine 6 was applied to aged mouse skin, it was found to increase the follicle number [52]. CASIN, which is a CDC42 inhibitor, also induced HFSC activation and hair growth by restoring the Wnt canonical pathway in aged mice [53].

Most research on HFSC rejuvenation has primarily focused on observations of improved hair growth in aged animal skin following treatment with target compounds or therapies. Studies investigating the underlying cellular signaling pathways responsible for inhibition of age-related hair loss are rare. We established an in vitro model of HFSC senescence and treated the senescent HFSCs with CM from PDLLA-treated macrophages. We discovered that PDLLA treatment enhances M2 macrophage polarization via Piezo1, which in turn regulates the HFSC niche. This regulation leads to maintenance of HFSC stemness, increased HFSC proliferation, and upregulation of various molecules that sustain anagen. Furthermore, the cellular signals upregulated by PDLLA were attenuated after treatment with a Piezo1 inhibitor, confirming the involvement of Piezo1 in PDLLA-induced M2 macrophage polarization. Despite these findings, a limitation of this study is the lack of specificity of the Piezo1 inhibitor that we used. Nevertheless, this study shows that PDLLA stimulates senescent HFSCs to progress into anagen. This finding highlights the potential of PDLLA to reverse age-induced stem cell alterations, offering a promising avenue for future therapies targeting age-related hair loss.

As our findings move toward potential clinical translation, several practical factors warrant attention. First, PDLLA belongs to the same family of biostimulatory fillers as poly-L-lactic acid (PLLA), which has been widely used and is approved in multiple regions for facial volume restoration. Clinical experience and prospective studies with PLLA have shown gradual collagen stimulation, predictable biodegradation, and an acceptable long-term safety profile when proper injection techniques are used [54]. Likewise, a 24-month multicenter study of PDLLA filler for nasolabial fold correction demonstrated sustained efficacy and a low rate of serious adverse events, supporting its suitability as an injectable dermal implant [55]. Nevertheless, case reports of granuloma formation after PDLLA injection indicate that delayed inflammatory reactions can occur and should be carefully monitored, particularly in the context of repeated or high-volume treatments [56]. Taken together, these data suggest that PDLLA-based materials are generally well tolerated in chronic cosmetic use, but dedicated clinical trials focusing on scalp administration will be required to fully establish long-term safety in hair-related indications. Second, PDLLA should be considered in the context of existing hair-regeneration strategies. Platelet-rich plasma (PRP) has shown overall favorable outcomes in androgenetic alopecia, but its efficacy can be variable and often depends on repeated sessions and operator-dependent preparation protocols [57]. Microneedling has also been reported to significantly improve hair density when combined with topical minoxidil in randomized, evaluator-blinded trials, highlighting the benefit of controlled micro-injury and wound-healing pathways [58]. More recently, exosome-based approaches, particularly those using adipose-derived stem cell exosomes, have emerged as promising paracrine therapies for hair regrowth, although regulatory frameworks and manufacturing standardization remain evolving [59,60]. In contrast, PDLLA provides a sustained biophysical and mechanotransductive stimulus that modulates the immune-stem cell niche over months rather than days or weeks. This durability may offer certain advantages in terms of effect duration, although it could also be accompanied by a slower onset of response compared with biologic approaches. Accordingly, PDLLA-based treatment may serve as one of several potential adjunctive options alongside PRP, microneedling, or exosome therapy, and future studies should determine whether combination strategies provide added benefit.

## Data Availability

All datasets generated or analyzed in this study are included in the article.

## Supplementary Materials

The following supporting information can be downloaded at: https://www.mdpi.com/article/10.3390/cells15010005/s1, Figure S1: H_2_O_2_ induces oxidative stress and senescence in THP-1 cells; Figure S2: PDLLA induces Piezo1 expression and calcium influx in senescent macrophages; Figure S3: Optimization of the H_2_O_2_-induced senescence model in HFSCs; Table S1: List of primers for quantitative real-time PCR; Table S2: List of antibodies used for Western blot, flow cytometry, ELISA, IF and IHC.

## Abbreviations

The following abbreviations are used in this manuscript:

AAALAC	Association for Assessment and Accreditation of Laboratory Animal Care
BMP	Bone morphogenetic protein
CM	Conditioned media
DP	Dermal papilla
DPBS	Dulbecco’s phosphate-buffered saline
ERK	Extracellular signal-regulated kinases
FBS	Fetal bovine serum
GSK3β	Glycogen synthase kinase 3β
HA	Hyaluronic acid
HFSCs	Hair follicle stem cells
HGF	Hepatocyte growth factor
IACUC	Institutional Animal Care and Use Committee
IGF-1	Insulin-like growth factor-1
JAK	Janus kinase
PCNA	Proliferating cell nuclear antigen
Piezo1	Piezo-type mechanosensitive ion channel component 1
PMA	Phorbol 12-myristate 13-acetate
P/S	Penicillin/streptomycin
PDLLA	Poly-D,L-lactic acid
PLLA	Poly-L-lactic acid
PRP	Platelet-rich plasma
SA-β-gal	Senescence-associated beta-galactosidase
TCF/LEF	T cell factor/lymphoid enhancer factor
TRPC	Transient receptor potential channel
TRPV	Transient receptor potential channel subfamily V member
8-OHdG	8-hydroxy-2′-deoxyguanosine
