# Supplementary material for: Poly-D,L-Lactic Acid Filler Increases Hair Growth by Modulating Hair Follicular Stem Cells in Aged Skin
Source: Cells. 2025 Dec 19;15(1):5. doi: 10.3390/cells15010005 (PMC12785112; doi:10.3390/cells15010005)
Supplement: Supplementary file 1 [file cells-15-00005-s001.zip › cells-4001365-supplementary.pdf]

cells

Article

Poly-D,L-Lactic Acid Filler Increases Hair Growth by Modulating Hair Follicular Stem Cells in Aged Skin

Seyeon Oh [1,2,†], Jino Kim [1,3,4,†], Hosung Choi [5], Hwa Jung Yoo [6], Kuk Hui Son [7,*] and Kyunghee Byun [1,3,8,*]

1 Functional Cellular Networks Laboratory, Lee Gil Ya Cancer and Diabetes Institute, Gachon University, Incheon 21999, Republic of Korea; seyeon8965@gmail.com (S.O.); jinokims@gmail.com (J.K.)
2 LIBON Inc., Incheon 22006, Republic of Korea
3 Department of Anatomy & Cell Biology, College of Medicine, Gachon University, Incheon 21936, Republic of Korea
4 New Hair Plastic Surgery Clinic, Seoul 06134, Republic of Korea
5 PIENA Aesthetic Medical Clinic, Seoul 06136, Republic of Korea; uroskin2121@gmail.com
6 MODI Hair Plant Clinic, Seoul 06729, Republic of Korea; modihairplant@gmail.com
7 Department of Thoracic and Cardiovascular Surgery, Gachon University Gil Medical Center, Gachon University, Incheon 21565, Republic of Korea
8 Department of Health Sciences and Technology, Gachon Advanced Institute for Health & Sciences and Technology (GAIHST), Gachon University, Incheon 21999, Republic of Korea
* Correspondence: dr632@gachon.ac.kr (K.H.S.); khbyun1@gachon.ac.kr (K.B.); Tel.: +82-32-460-3666 (K.H.S.); +82-32-899-6511 (K.B.)
† These authors contributed equally to this study.

Academic Editor: Hideyuki J. Majima

Received: 6 November 2025
Revised: 12 December 2025
Accepted: 16 December 2025
Published: 19 December 2025

Copyright: © 2025 by the authors. Licensee MDPI, Basel, Switzerland. This article is an open access article distributed under the terms and conditions of the Creative Commons Attribution (CC BY) license.

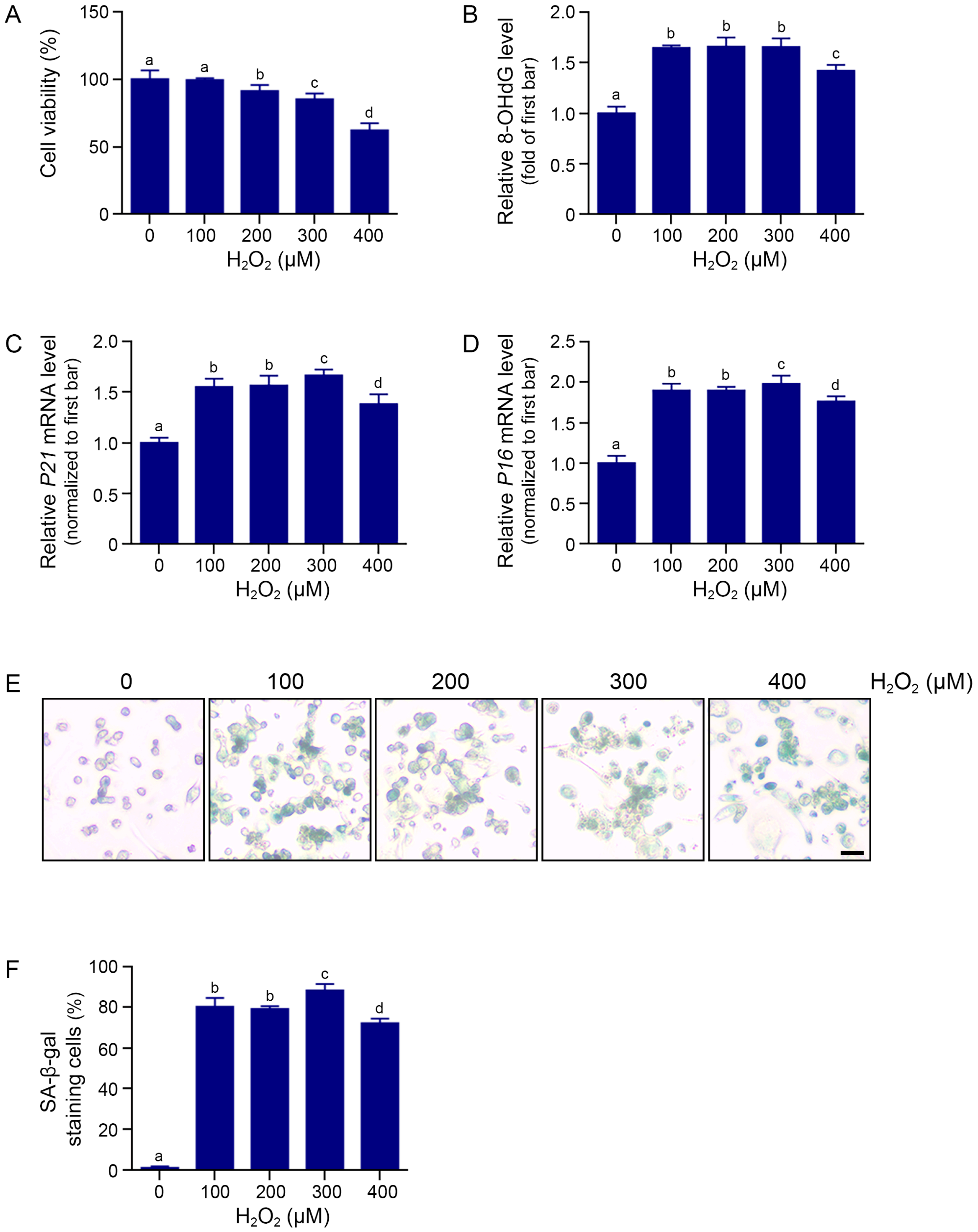

Figure S1. H_2O_2 induces oxidative stress and senescence in THP-1 cells. (**A**) Cell viability was measured by CCK-8 assay after 3 h of treatment with various concentrations of H_2O_2 (0–400 μM), followed by 72 h incubation in fresh growth medium; (**B**) Oxidative DNA damage was assessed by quantifying 8-hydroxy-2′-deoxyguanosine (8-OHdG) levels using ELISA; (**C**,**D**) mRNA expression levels of senescence-associated markers *P21* (**C**) and *P16* (**D**) were quantified by quantitative RT-PCR and normalized to *ACTB*. Data are presented as the fold change relative to the untreated control (0 μM; first bar); (**E**) Representative images of senescence-associated β-galactosidase (SA-β-gal) staining. Blue color indicates SA-β-gal–positive cells. Scale bar = 30 μm; (**F**) Quantification of SA-β-gal–positive cells per field. Data are presented as the mean ± SD. Statistical significance was determined by Kruskal–Wallis test followed by pairwise Mann–Whitney U test. Different letters above bars indicate statistically significant differences between groups ($p < 0.05$).

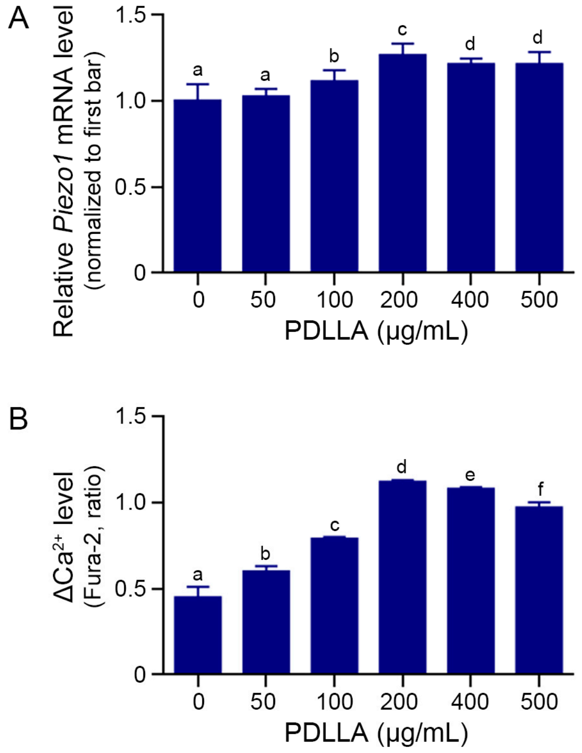

Figure S2. PDLLA induces Piezo1 expression and calcium influx in senescent macrophages. (**A**) Relative mRNA expression levels of *Piezo1* were measured by quantitative RT-PCR after 48 h treatment with various concentrations of PDLLA (0–500 μg/mL). Expression levels were normalized to *ACTB* and presented as the fold change relative to the untreated control (0μg/mL; first bar); (**B**) Intracellular Ca^{2+} influx was evaluated by Fura-2 radiometric fluorescence after 2 h of PDLLA treatment. Changes in calcium levels ($\Delta[Ca^{2+}]$) were calculated as the difference in fluorescence ratio (340/380 nm) after PDLLA stimulation. Data are presented as the mean ± SD. Statistical significance was determined by Kruskal–Wallis test followed by pairwise Mann–Whitney U test. Different letters indicate significant differences between groups ($p < 0.05$).

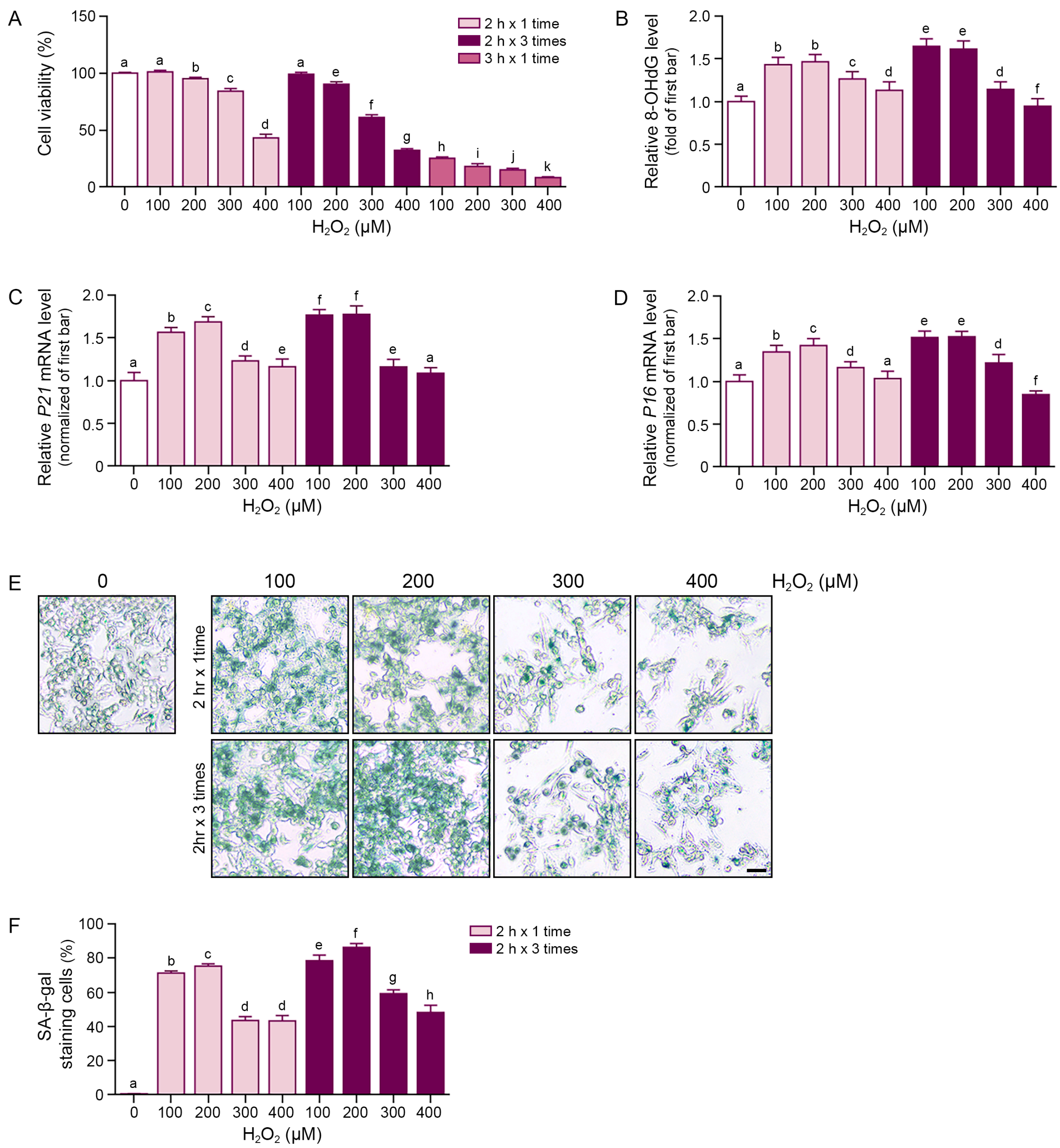

Figure S3. Optimization of the H_2O_2-induced senescence model in HFSCs. (**A**) Cell viability was assessed after treatment with different concentrations of H_2O_2 (0–400 μM) for 2 h (2 h x 1 time), 3 h (3 h x 1 time), or three repeated 2 h exposures at 2 h intervals (2 h x 3 times); (**B**) Oxidative DNA damage was evaluated by measuring 8-OHdG levels in HFSCs after 2 h x 1 time or 2 h x 3 times H_2O_2 exposure; (**C**,**D**) mRNA expression levels of senescence markers *P21* (**C**) and *P16* (**D**) were analyzed by quantitative RT-PCR and normalized to *ACTB*. Data are presented as the fold change relative to the untreated control (0 μM; first bar); (**E**) Representative images of senescence-associated β-galactosidase (SA-β-gal) staining showing senescent HFSCs exposed to 0–400 μM H_2O_2 for 2 h x 1 time (top row) or 2 h x 3 times (bottom row). Blue color indicates SA-β-gal–positive cells. Scale bar = 30 μm. (**F**) Quantification of SA-β-gal–positive cells per field. Data are presented as the mean ± SD. Statistical significance was determined by Kruskal–Wallis test followed by pairwise Mann–Whitney U test. Different letters indicate significant differences between groups ($p < 0.05$).

Table S1. List of primers for quantitative real-time PCR.

Gene *(Organism)*	Primer sequences	
ACTB (human)	Forward	5'-GGG ACC TGA CTG ACT ACC TCA T-3′
	Reverse	5'-CCT TAA TGT CAC GCA CGA TTT-3′
P16 (human)	Forward	5'-GGC CTT CTT CCT CTT CTG CT-3′
	Reverse	5'-GCA ACA TCA CCA ATG GAC AG-3′
P21 (human)	Forward	5'-ATA TCA GGA AAA AGG GTG CAG-3′
	Reverse	5'-CAG AAT GAG GAA CTC CTG GAA G-3′
Piezo1 (human)	Forward	5'-ACA GAC ATC ACG TCC TCC CTA T-3′
	Reverse	5'-TAC TGA ACT GGA TCA GCA GCA T-3′

Table S2. List of antibodies used for Western blot, flow cytometry, ELISA, IF and IHC.

Antibody	Dilution rate				
	Western blot	Flow cytometry	ELISA	IF	IHC
**Piezo1** (Proteintech, #15939-1-AP)	1:500				
**β-actin** (Cell signaling, #4967)	1:1,000				
**CD86** (Santa Cruz Biotechnology, #sc-19617)		1:100			1:100
**CD206** (Novus Biogicals LLC, #NBP1-90020)		1:100			1:100
**HGF** (Bioss, #bs-1025R)		1:500			
**IGF-1** (Bioss, bs-0014R)		1:500			
**RAS** (Bioss, bs-1033R)	1:500				
**pERK1/2 (Thr202/Tyr204)** (Cell signaling, #9101)	1:1,000				
**ERK1/2** (Cell signaling, #9102)	1:1,000				
**c-FOS** (Cell signaling, #2250)	1:500				
**K15** (Invitrogen, #MA5-11344)	1:500			1:200	
**PCNA** (abcam, #ab18197)	1:1,000			1:1,000	
**Wnt3a** (Bioss, bs-1700R)	1:1,000				
**Wnt10b** (Bioss, bs-3662R)	1:1,000				
**pGSK3β (Ser9)** (Cell signaling, #5558)	1:1,000				
**GSK3β** (Cell signaling, #9315)	1:1,000				
**β-catenin** (Santa Cruz Biotechnology, #sc-7963)	1:500				
**Histone H3** (Cell signaling, #9715)	1:1,000				
**Axin2** (Cusabio, #CSB-PA917071)	1:500				1:100
**Lef1** (Santa Cruz Biotechnology, #sc-374522)	1:1,000				1:200

**Lgr5**<br>(Bosterbio, #A00239-2)	1:500				1:100
**8-OHdG**<br>(Abcam, #ab62623)			1:500		
